# Supplementary material for: Proteomic Analysis of Dhh1 Complexes Reveals a Role for Hsp40 Chaperone Ydj1 in Yeast P-Body Assembly
Source: G3 (Bethesda). 2015 Sep 21;5(11):2497–511. doi: 10.1534/g3.115.021444 (PMC4632068; doi:10.1534/g3.115.021444)
Supplement: Supporting Information [file supp_5_11_2497__index.html]

Proteomic Analysis of Dhh1 Complexes Reveals a Role for Hsp40 Chaperone Ydj1 in Yeast P-Body Assembly — Supporting Information 

# Proteomic Analysis of Dhh1 Complexes Reveals a Role for Hsp40 Chaperone Ydj1 in Yeast P-Body Assembly

## Supporting Information for Cary *et al.*, 2015

**Files in this Data Supplement:**

- Supporting Information - Figures S1-S6 and Tables S1-S8 (PDF, 1 MB)
- Figure S1 - Results from two replicate I-DIRT experiments. (PDF, 516 KB)
- Figure S2 - Venn diagrams of all proteins identified across the replicates and conditions. (PDF, 520 KB)
- Figure S3 - Microscopic images of Dhh1-GFP induction in wild type and mutant strains *hsp104Δ*, two Hsp70 mutants (*ssa1Δ* and *ssa2Δ*), and two Hsp90 mutants (*hsc82Δ* and *hsp82Δ*). (PDF, 541 KB)
- Figure S4 - Protein levels in *ydj1Δ* mutant strains. (PDF, 548 KB)
- Figure S5 - Quantitative reverse transcriptase PCR assessment of enriched transcripts. (PDF, 558 KB)
- Figure S6 - Protein and RNA co-enrichment. (PDF, 574 KB)
- Table S1 - *S. cerevisiae* strains used in this study. (PDF, 77 KB)
- Table S2 - Protein shortlist based on proteomic observation of Dhh1-GFP immunoprecipitations. (.xlsx, 50 KB)
- Table S3 - I-DIRT data for proteins in proteomics shortlist. (.xlsx, 53 KB)
- Table S4 - Dhh1-GFP interacting proteins annotated as RNA-binding proteins (RBP). (.xlsx, 15 KB)
- Table S5 - Relative protein abundance measured by normalized spectral counts. (.xlsx, 43 KB)
- Table S6 - Dhh1-GFP interacting proteins that respond to various stress factors. (.xlsx, 86 KB)
- Table S7 - Prediction and characterization of low-complexity proteins. (.xlsx, 57 KB)
- Table S8 - Transcripts identified by Dhh1-GFP immunoprecipitation experiments. (.xlsx, 13 KB)
