# Supplementary material for: Proteomic Analysis of Dhh1 Complexes Reveals a Role for Hsp40 Chaperone Ydj1 in Yeast P-Body Assembly
Source: G3 (Bethesda). 2015 Sep 21;5(11):2497–511. doi: 10.1534/g3.115.021444 (PMC4632068; doi:10.1534/g3.115.021444)
Supplement: Supporting Information [file supp_g3.115.021444_021444SI.pdf]

G3: Genes | Genomes | Genetics

# Proteomic analysis of Dhh1 complexes reveals a role for Hsp40 chaperone Ydj1 in yeast P-body assembly

Gregory A. Cary[1,2,5,6], Dani B.N. Vinh[1,5,7], Patrick May[1,3], Rolf Kuestner[1,8], Aimée M. Dudley[2,4]*

[1]Institute for Systems Biology, Seattle WA

[2]Molecular and Cellular Biology Program, , Seattle WA

[3]Luxembourg Centre for Systems Biomedicine, Luxembourg

[4]Pacific Northwest Diabetes Research Institute, Seattle, WA

[5]Equally contributing author

*Correspondence: aimee.dudley@gmail.com

**DOI: 10.1534/g3.115.021444**

**Table S1.** *S. cerevisiae* strains used in this study.

| Strain | Genotype | Reference |
|---|---|---|
| BY4741 | MATa *his3Δ1 leu2Δ0 met15Δ0 ura3Δ0* | (Winston *et al.* 1995) |
| YAD49 | MATa *his3Δ1 leu2Δ0 met15Δ0 ura3Δ0 DHH1-GFP::HIS3MX6* | (Huh *et al.* 2003) |
| YAD50 | MATa *his3Δ1 leu2Δ0 met15Δ0 ura3Δ0 EDC3-GFP::HIS3MX6* | (Huh *et al.* 2003) |
| YAD52 | MATa *his3Δ1 leu2Δ0 met15Δ0 ura3Δ0 LSM1-GFP::HIS3MX6* | (Huh *et al.* 2003) |
| YAD53 | MATa *his3Δ1 leu2Δ0 met15Δ0 ura3Δ0 PAT1-GFP::HIS3MX6* | (Huh *et al.* 2003) |
| YAD557 | MATa *his3Δ1 leu2Δ0 met15Δ0 ura3Δ0 DHH1-GFP::HIS3MX6 ydj1Δ::kanMX* | This study |
| YAD591 | MATa *his3Δ1 leu2Δ0 met15Δ0 ura3Δ0 DHH1-GFP::HIS3MX6 hsp104Δ::kanMX* | This study |
| YAD553 | MATa *his3Δ1 leu2Δ0 met15Δ0 ura3Δ0 DHH1-GFP::HIS3MX6 ssa1Δ::kanMX* | This study |
| YAD554 | MATa *his3Δ1 leu2Δ0 met15Δ0 ura3Δ0 DHH1-GFP::HIS3MX6 ssa2Δ::kanMX* | This study |
| YAD556 | MATa *his3Δ1 leu2Δ0 met15Δ0 ura3Δ0 DHH1-GFP::HIS3MX6 hsp82Δ::kanMX* | This study |
| YAD555 | MATa *his3Δ1 leu2Δ0 met15Δ0 ura3Δ0 DHH1-GFP::HIS3MX6 hsc82Δ::kanMX* | This study |
| YAD559 | MATa *his3Δ1 leu2Δ0 met15Δ0 ura3Δ0 LSM1-GFP::HIS3MX6 ydj1Δ::kanMX* | This study |
| YAD561 | MATa *his3Δ1 leu2Δ0 met15Δ0 ura3Δ0 EDC3-GFP::HIS3MX6 ydj1Δ::kanMX* | This study |
| YAD393 | MATa *his3Δ1 leu2Δ0 met15Δ0 ura3Δ0* + p413-TEF-GFP(S65T) | This study |

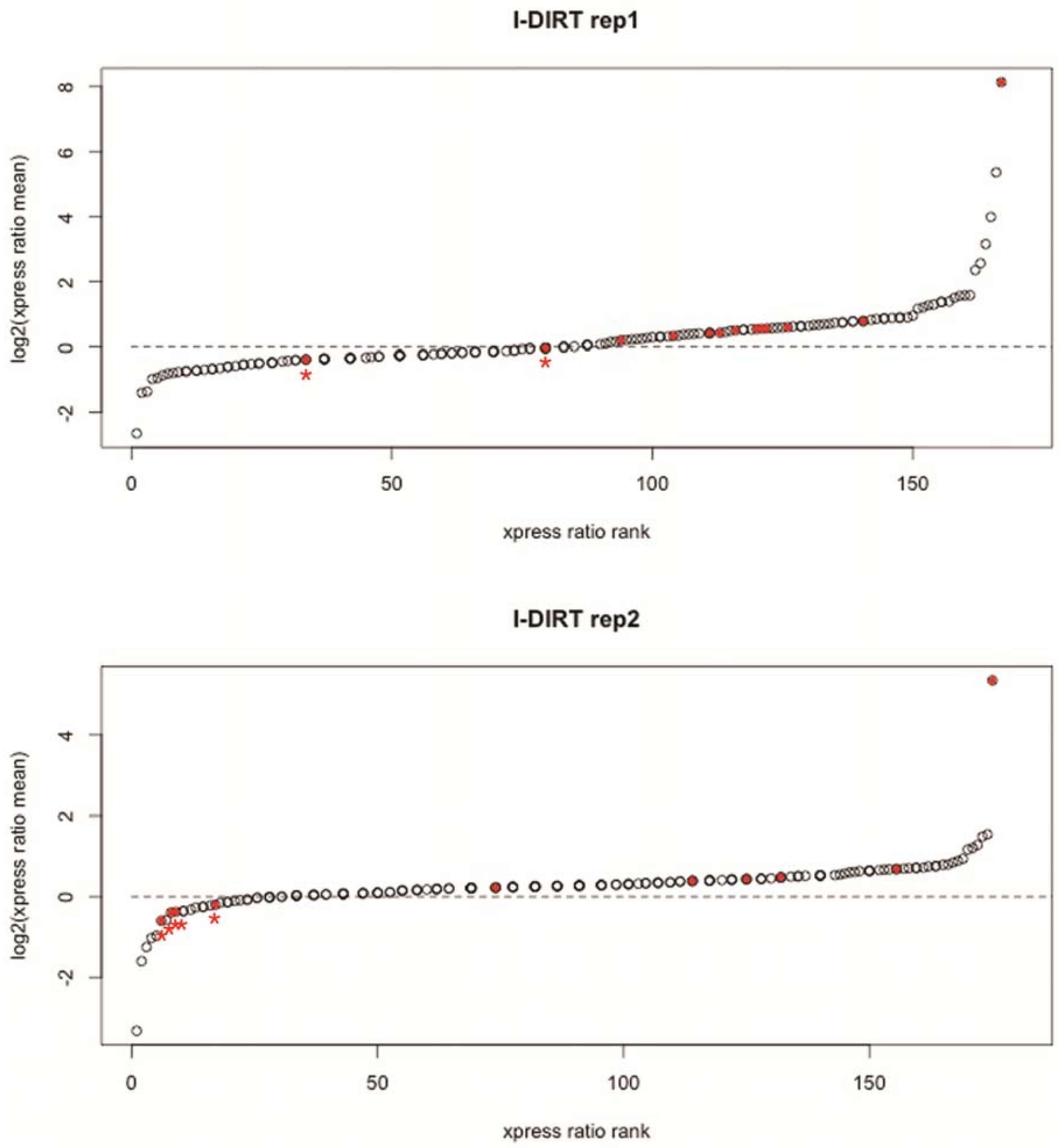


**Figure S1. Results from two replicate I-DIRT experiments.** The log-transformed average xpress ratio of light:heavy for all peptides from all proteins identified in the two Dhh1-GFP IP replicate samples plotted against the rank of the ratio. The dotted line corresponds to a 50:50 ratio of light:heavy peptides for given proteins. Known P-body proteins are colored in red. In each case, the known P-body components detected at <50:50 light:heavy are marked with an asterisk.

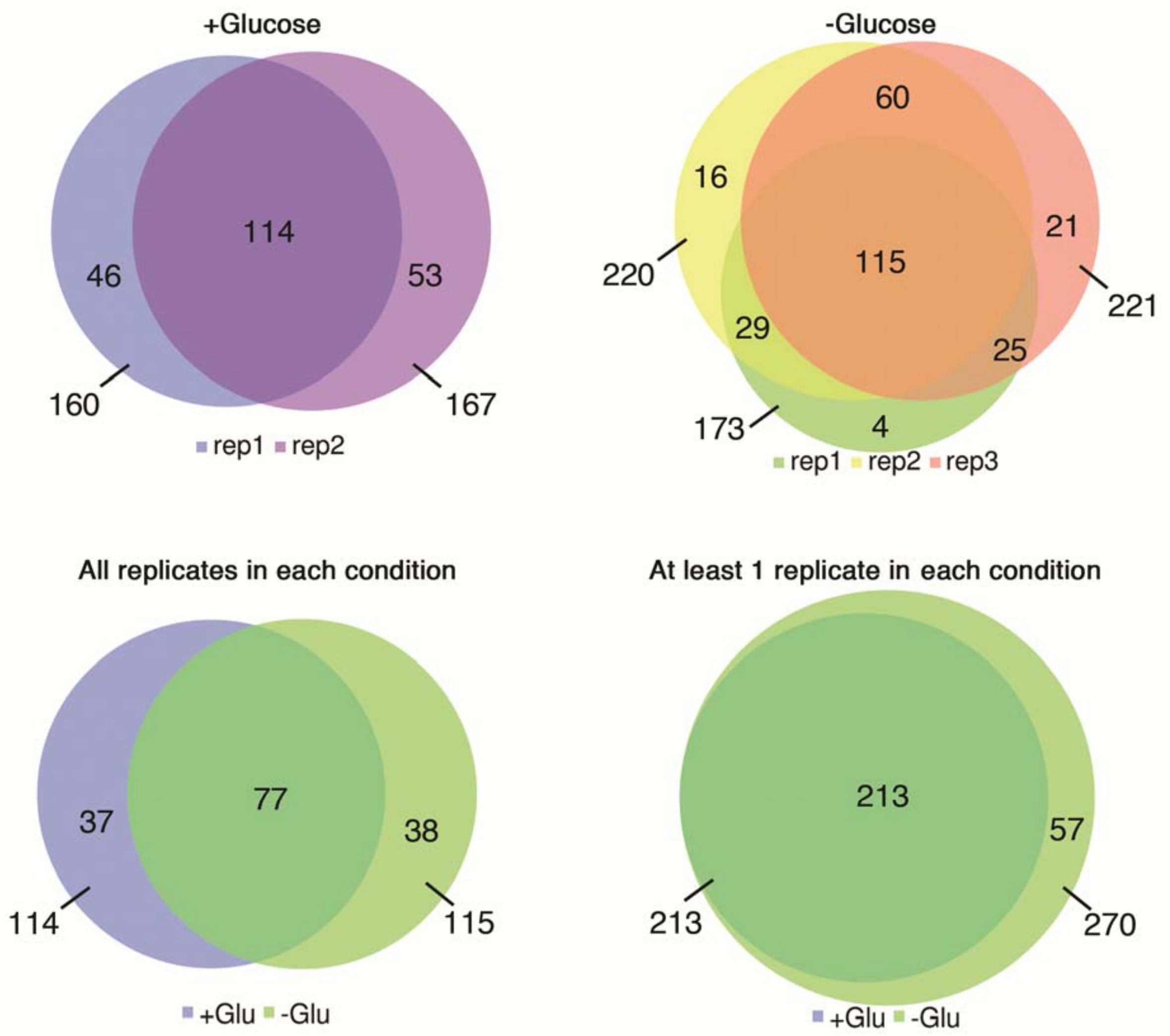


**Figure S2. Venn diagrams of all proteins identified across the replicates and conditions.** For each circle, the total number of proteins in that set is indicated (with connecting lines), as are the numbers of proteins within each overlapping set. All replicates in each condition represents the complete intersect of all replicates; at least one replicate in each condition is the union of all replicates from each condition.

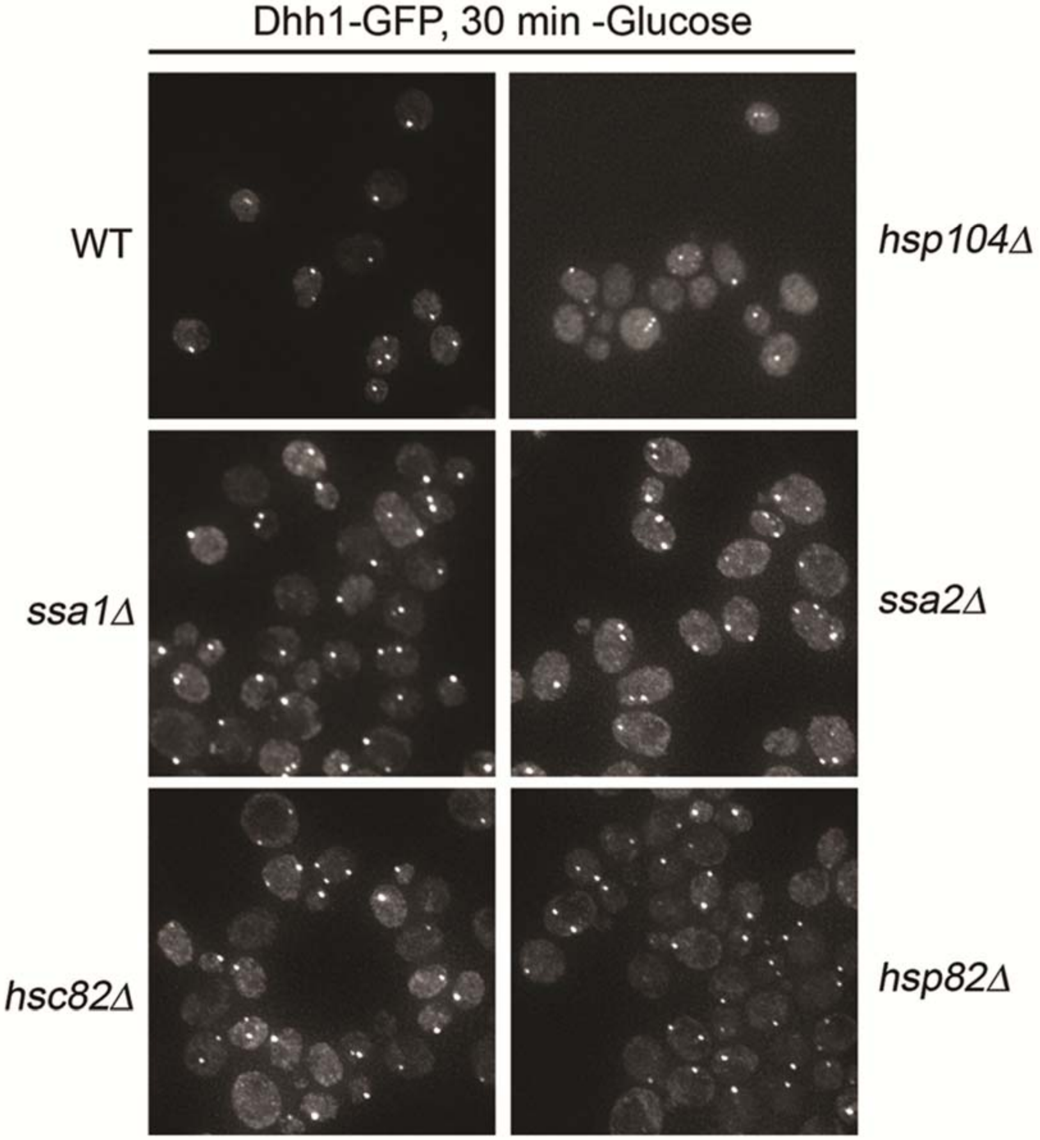


**Figure S3. Microscopic images of Dhh1-GFP induction in wild type and mutant strains *hsp104Δ*, two Hsp70 mutants (*ssa1Δ* and *ssa2Δ*), and two Hsp90 mutants (*hsc82Δ* and *hsp82Δ*).** Cells were induced to form foci by 30 minutes of glucose depletion.

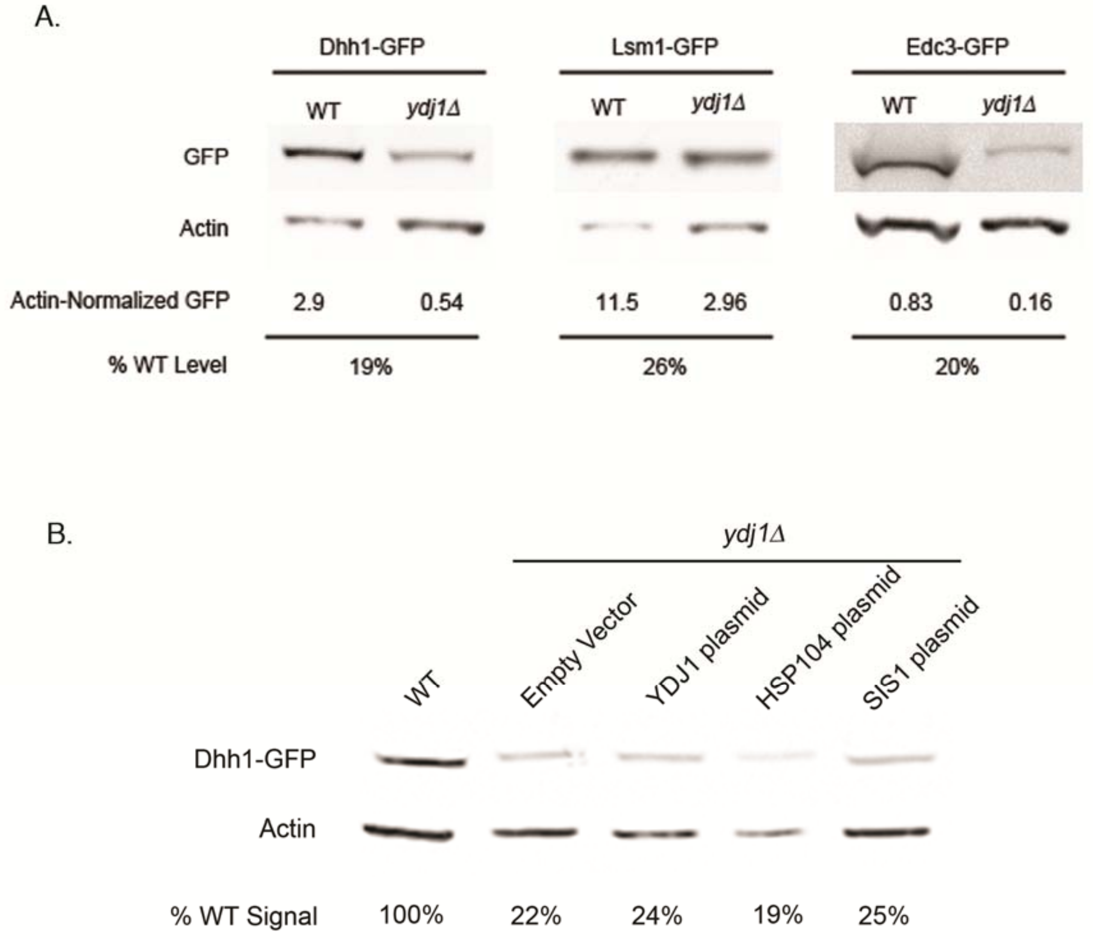


**Figure S4. Protein levels in *ydj1Δ* mutant strains.** (A) Anti-GFP western blots for Dhh1-GFP, Lsm1-GFP, and Edc3-GFP in WT and *ydj1Δ* mutant strain backgrounds. Anti-GFP signal is normalized to anti-Actin signal from the same lysate. Two independent isolates of the *ydj1Δ* background were tested for each protein and each yielded similar decreases in protein levels. (B) Anti-GFP western blot from Dhh1-GFP expressing strains either WT or *ydj1Δ* transformed with plasmids shown, either an empty vector or MoBY plasmids expressing the HSP indicated. For each lysate, Dhh1-GFP levels were normalized to anti-Actin levels in the same lysate and compared to WT levels.

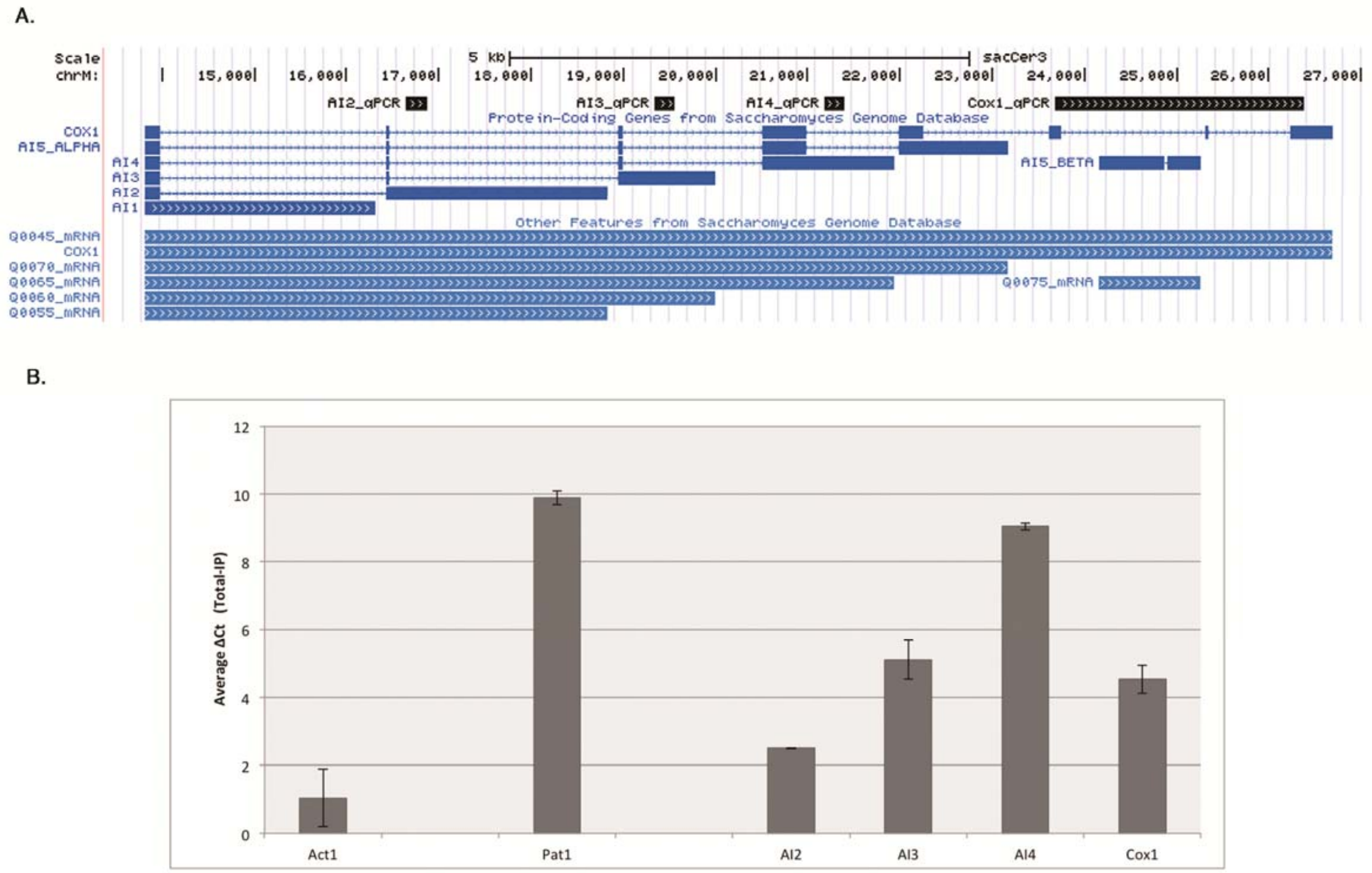


**Figure S5. Quantitative reverse transcriptase PCR assessment of enriched transcripts.** (A) Diagram of the mitochondrial *COX1* locus showing the locations of each primer set used to detect the various transcript splice forms present. (B) RT-qPCR results for genes identified as enriched by microarray assessment of Dhh1-GFP IP. The average delta Ct of each probe set between the Total and The IP sample is reported and represents three replicate measurements of the same IP experiment. *Act1* is shown as a negative control and *Pat1* is shown as a positive control (strongly enriched in each IP). Primer sets shown are specific to one group II intron (AI2), two group I introns (AI3 and AI4), and the primers to the *Cox1* transcript are intron-spanning so that only the spliced transcript would produce a signal.

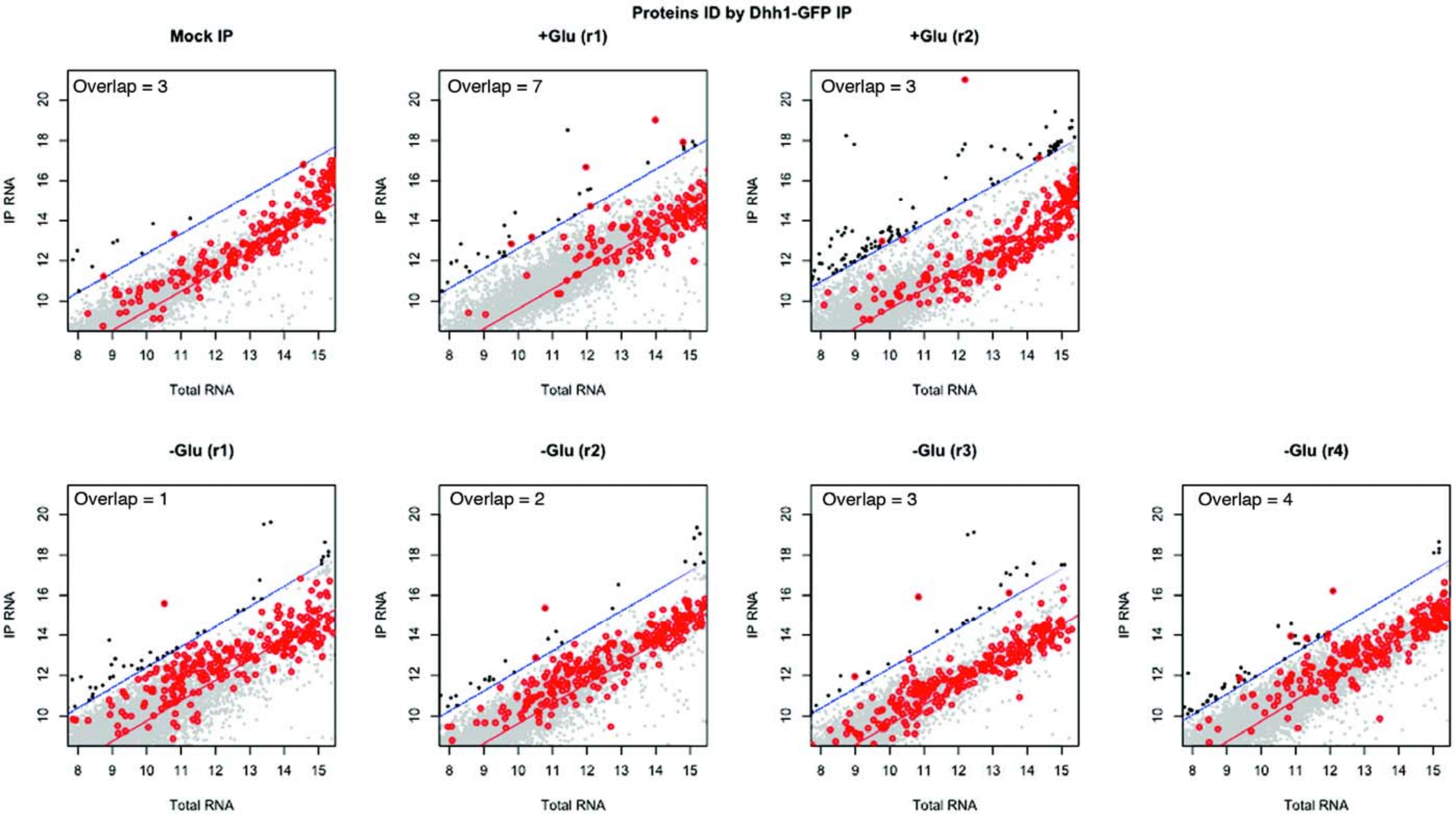


**Figure S6. Protein and RNA co-enrichment.** The transcripts encoding the 329 proteins identified in the proteomics dataset are circled in red. For each immuno-isolation of Dhh1-GFP, the overlap between enriched transcripts and the total number of all proteins identified is reported.

**Tables S2-S8**

Available for download at www.g3journal.org/lookup/suppl/doi:10.1534/g3.115.021444/-/DC1

**Table S2** Protein shortlist based on proteomic observation of Dhh1-GFP immunoprecipitations.

**Table S3** I-DIRT data for proteins in proteomics shortlist.

**Table S4** Dhh1-GFP interacting proteins annotated as RNA-binding proteins (RBP)

**Table S5** Relative protein abundance measured by normalized spectral counts.

**Table S6** Dhh1-GFP interacting proteins that respond to various stress factors

**Table S7** Prediction and characterization of low-complexity proteins.

**Table S8** Transcripts identified by Dhh1-GFP immunoprecipitation experiments.
