# Supplementary material for: Proteomic Analysis of Dhh1 Complexes Reveals a Role for Hsp40 Chaperone Ydj1 in Yeast P-Body Assembly
Source: G3 (Bethesda). 2015 Sep 21;5(11):2497–511. doi: 10.1534/g3.115.021444 (PMC4632068; doi:10.1534/g3.115.021444)
Supplement: Supporting Information [file supp_g3.115.021444_FigureS1.pdf]

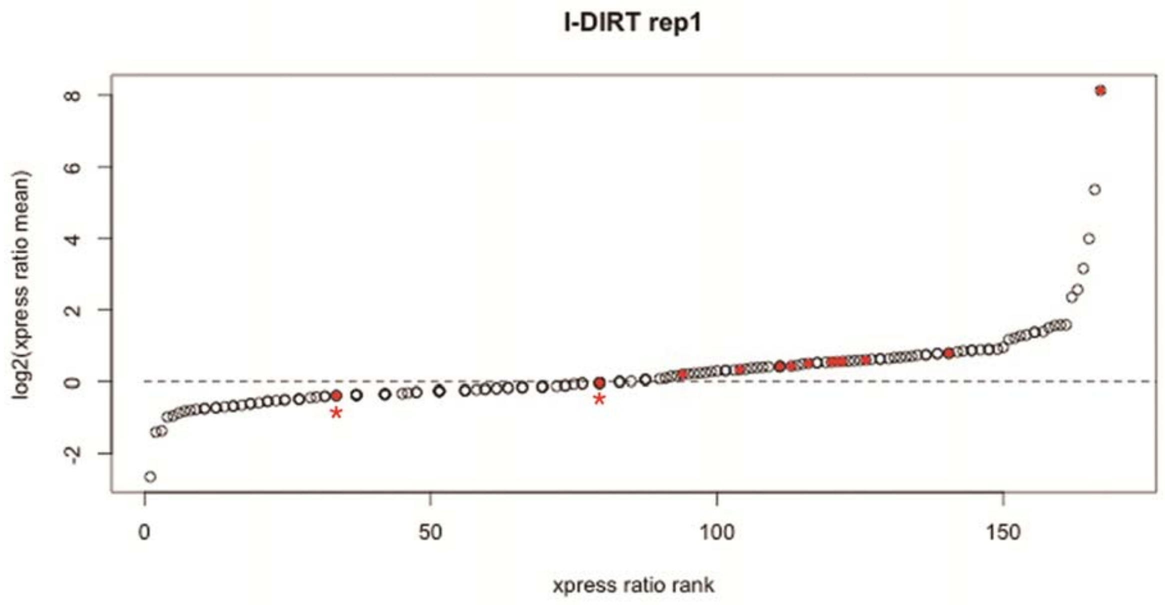

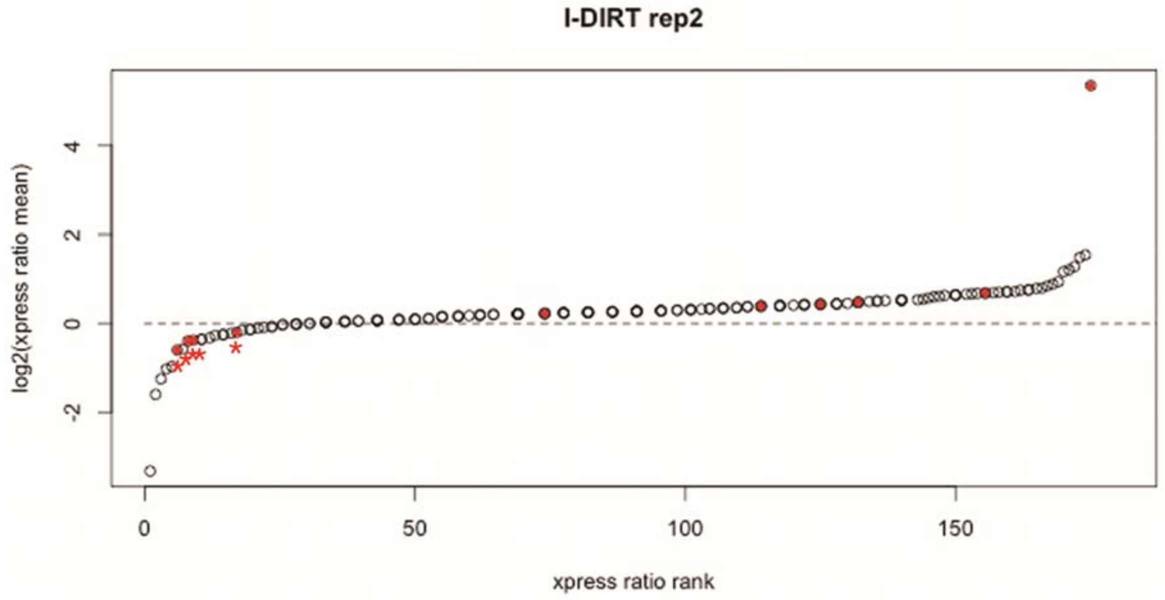

Figure S1. Results from two replicate I-DIRT experiments. The log-transformed average xpress ratio of light:heavy for all peptides from all proteins identified in the two Dhh1-GFP IP replicate samples plotted against the rank of the ratio. The dotted line corresponds to a 50:50 ratio of light:heavy peptides for given proteins. Known P-body proteins are colored in red. In each case, the known P-body components detected at <50:50 light:heavy are marked with an asterisk.
