# Supplementary material for: Proteomic Analysis of Dhh1 Complexes Reveals a Role for Hsp40 Chaperone Ydj1 in Yeast P-Body Assembly
Source: G3 (Bethesda). 2015 Sep 21;5(11):2497–511. doi: 10.1534/g3.115.021444 (PMC4632068; doi:10.1534/g3.115.021444)
Supplement: Supporting Information [file supp_g3.115.021444_FigureS2.pdf]

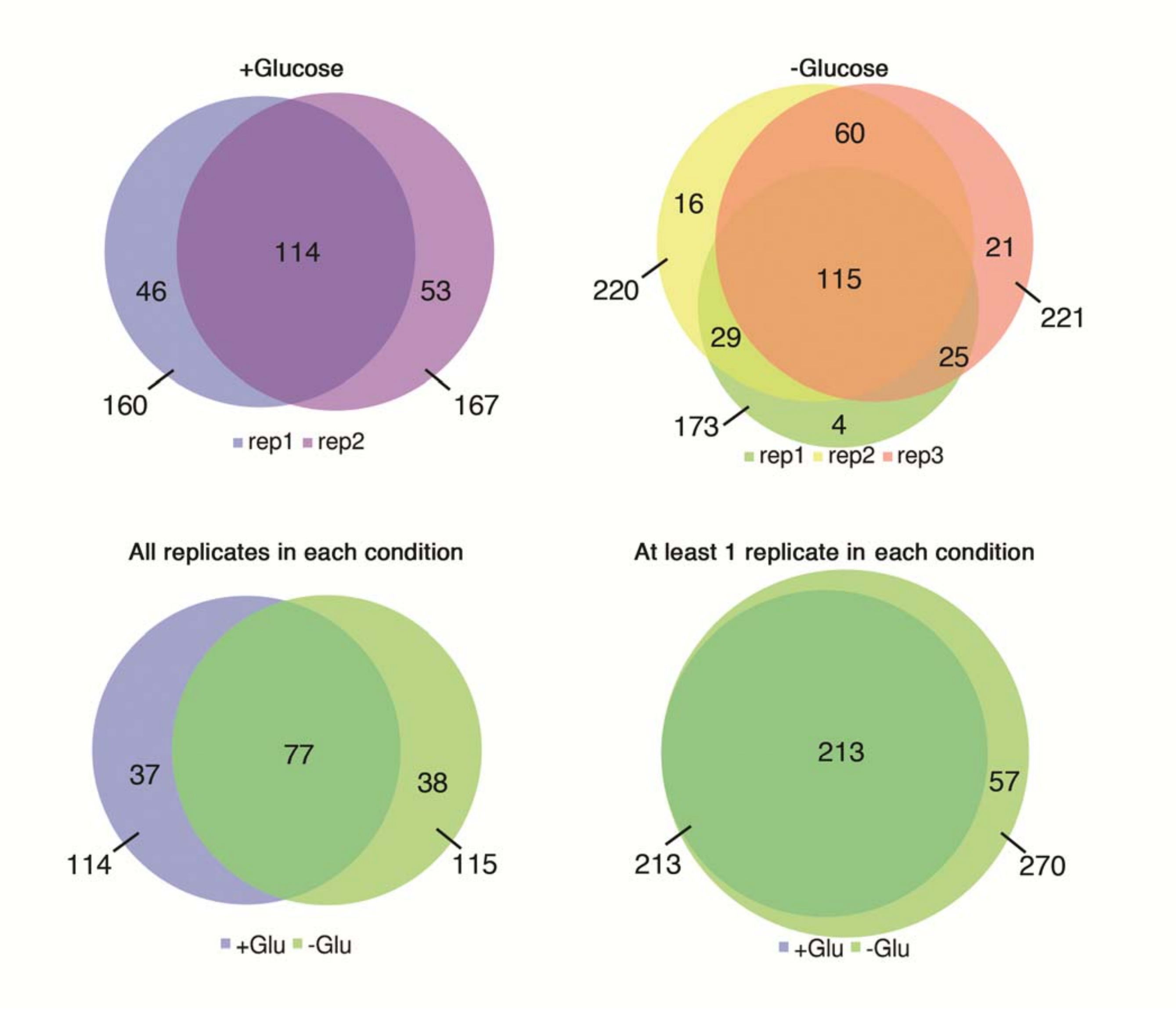

Figure S2. Venn diagrams of all proteins identified across the replicates and conditions. For each circle, the total number of proteins in that set is indicated (with connecting lines), as are the numbers of proteins within each overlapping set. All replicates in each condition represents the complete intersect of all replicates; at least one replicate in each condition is the union of all replicates from each condition.
