# Supplementary material for: Proteomic Analysis of Dhh1 Complexes Reveals a Role for Hsp40 Chaperone Ydj1 in Yeast P-Body Assembly
Source: G3 (Bethesda). 2015 Sep 21;5(11):2497–511. doi: 10.1534/g3.115.021444 (PMC4632068; doi:10.1534/g3.115.021444)
Supplement: Supporting Information [file supp_g3.115.021444_FigureS3.pdf]

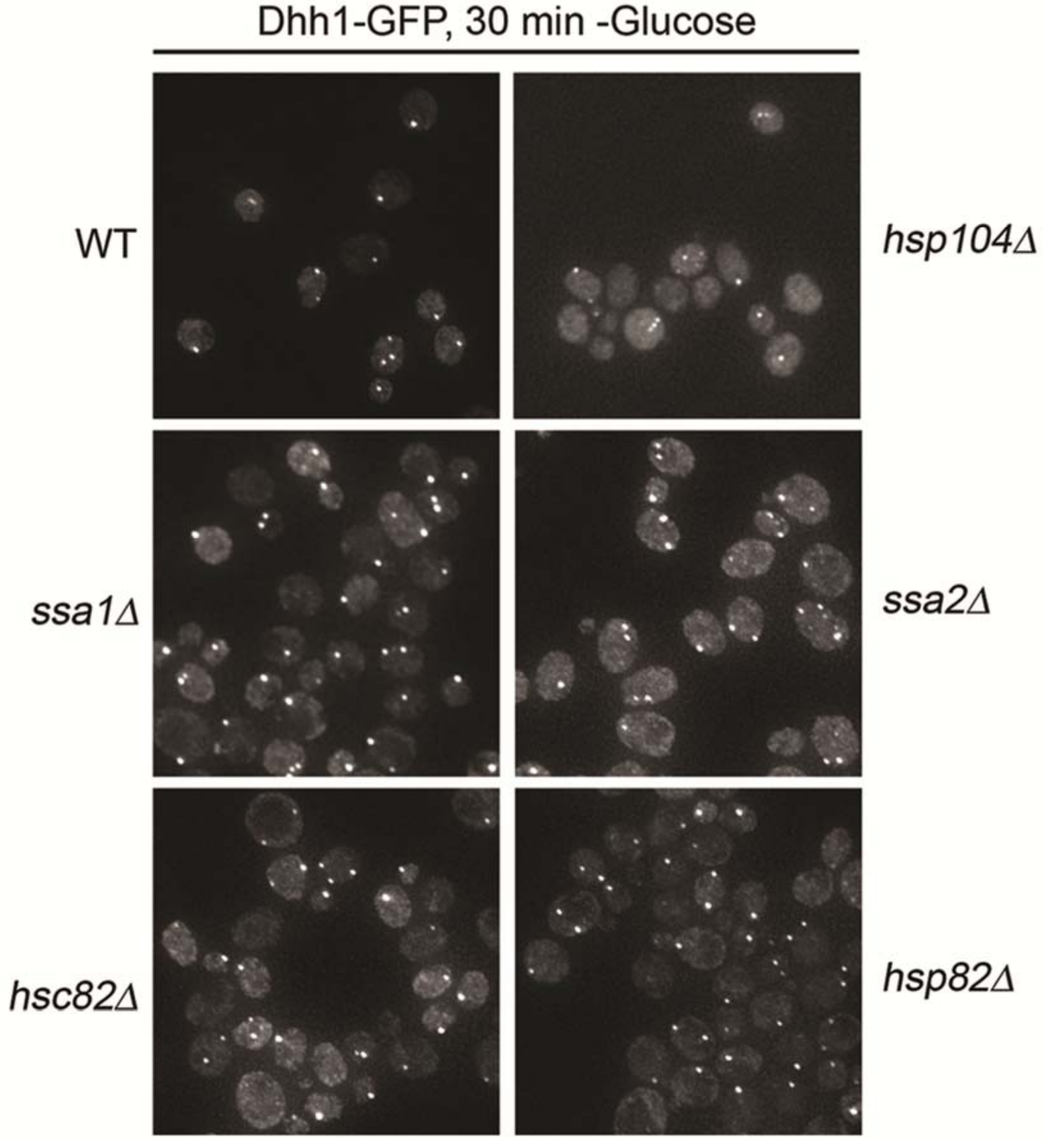

Figure S3. Microscopic images of Dhh1-GFP induction in wild type and mutant strains *hsp104Δ*, two Hsp70 mutants (*ssa1Δ* and *ssa2Δ*), and two Hsp90 mutants (*hsc82Δ* and *hsp82Δ*). Cells were induced to form foci by 30 minutes of glucose depletion.
