# Supplementary material for: Proteomic Analysis of Dhh1 Complexes Reveals a Role for Hsp40 Chaperone Ydj1 in Yeast P-Body Assembly
Source: G3 (Bethesda). 2015 Sep 21;5(11):2497–511. doi: 10.1534/g3.115.021444 (PMC4632068; doi:10.1534/g3.115.021444)
Supplement: Supporting Information [file supp_g3.115.021444_FigureS4.pdf]

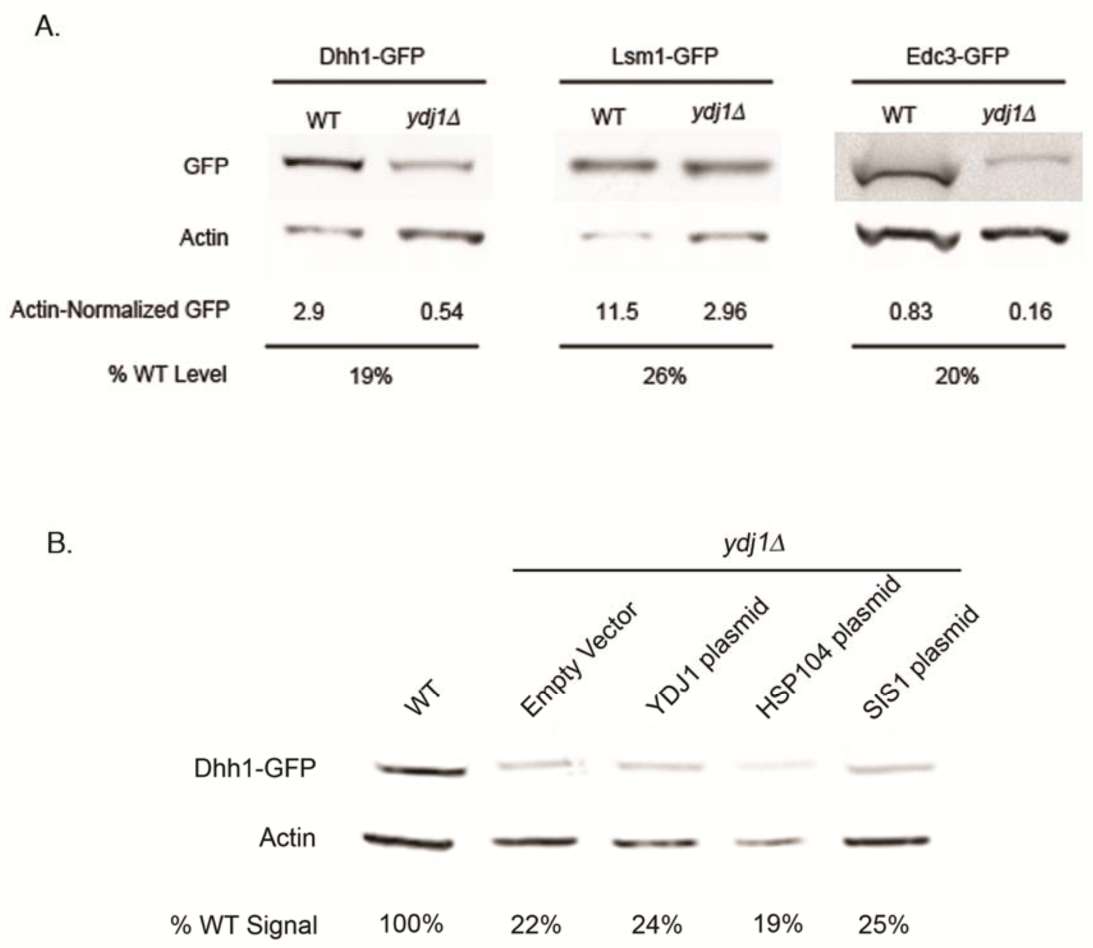

Figure S4. Protein levels in *ydj1Δ* mutant strains. (A) Anti-GFP western blots for Dhh1-GFP, Lsm1-GFP, and Edc3-GFP in WT and *ydj1Δ* mutant strain backgrounds. Anti-GFP signal is normalized to anti-Actin signal from the same lysate. Two independent isolates of the *ydj1Δ* background were tested for each protein and each yielded similar decreases in protein levels. (B) Anti-GFP western blot from Dhh1-GFP expressing strains either WT or *ydj1Δ* transformed with plasmids shown, either an empty vector or MoBY plasmids expressing the HSP indicated. For each lysate, Dhh1-GFP levels were normalized to anti-Actin levels in the same lysate and compared to WT levels.
