# Supplementary material for: Proteomic Analysis of Dhh1 Complexes Reveals a Role for Hsp40 Chaperone Ydj1 in Yeast P-Body Assembly
Source: G3 (Bethesda). 2015 Sep 21;5(11):2497–511. doi: 10.1534/g3.115.021444 (PMC4632068; doi:10.1534/g3.115.021444)
Supplement: Supporting Information [file supp_g3.115.021444_FigureS5.pdf]

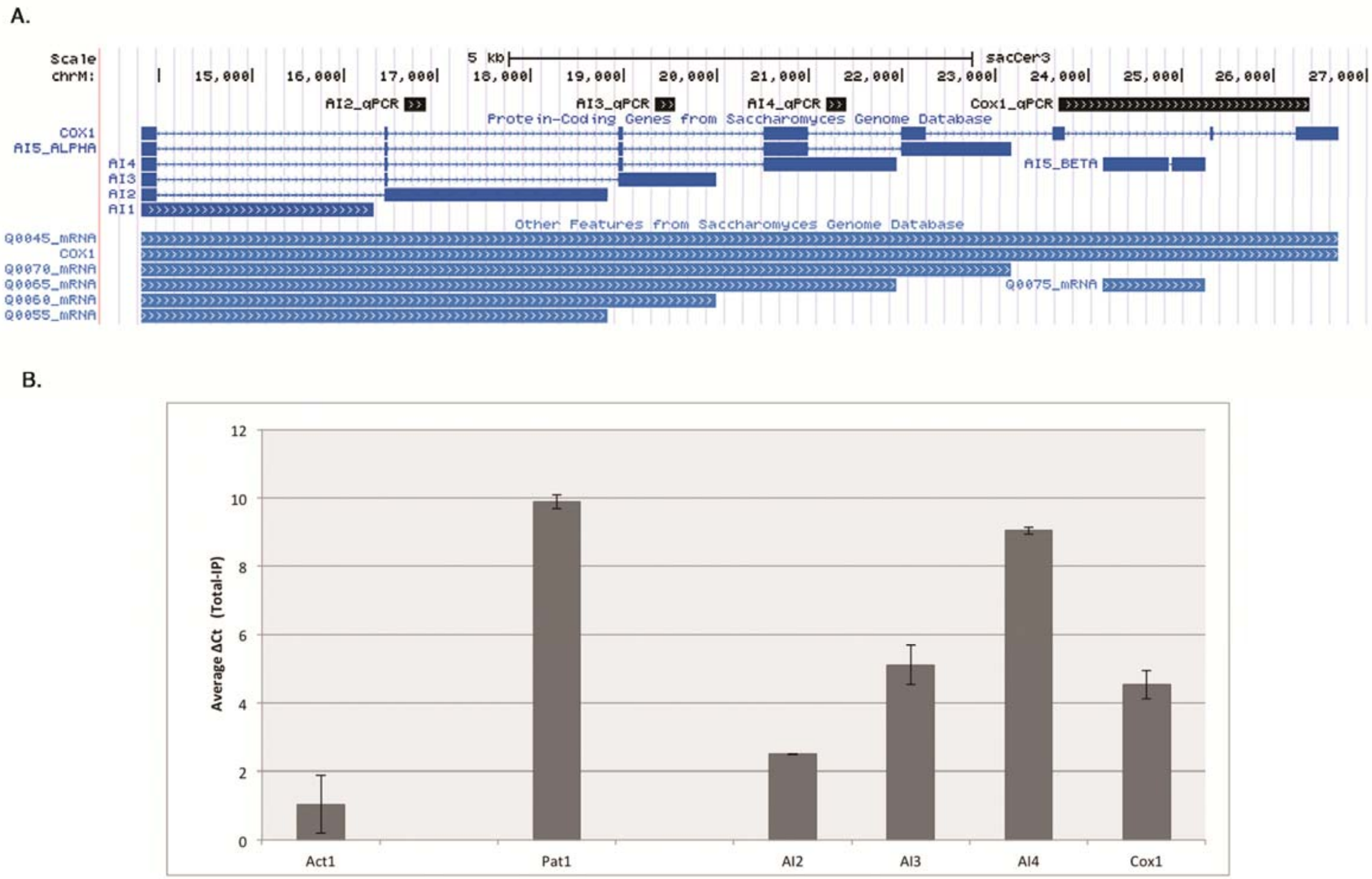

Figure S5. Quantitative reverse transcriptase PCR assessment of enriched transcripts. (A) Diagram of the mitochondrial *COX1* locus showing the locations of each primer set used to detect the various transcript splice forms present. (B) RT-qPCR results for genes identified as enriched by microarray assessment of Dhh1-GFP IP. The average delta Ct of each probe set between the Total and The IP sample is reported and represents three replicate measurements of the same IP experiment. *Act1* is shown as a negative control and *Pat1* is shown as a positive control (strongly enriched in each IP). Primer sets shown are specific to one group II intron (AI2), two group I introns (AI3 and AI4), and the primers to the *Cox1* transcript are intron-spanning so that only the spliced transcript would produce a signal.
