# Supplementary material for: Proteomic Analysis of Dhh1 Complexes Reveals a Role for Hsp40 Chaperone Ydj1 in Yeast P-Body Assembly
Source: G3 (Bethesda). 2015 Sep 21;5(11):2497–511. doi: 10.1534/g3.115.021444 (PMC4632068; doi:10.1534/g3.115.021444)
Supplement: Supporting Information [file supp_g3.115.021444_FigureS6.pdf]

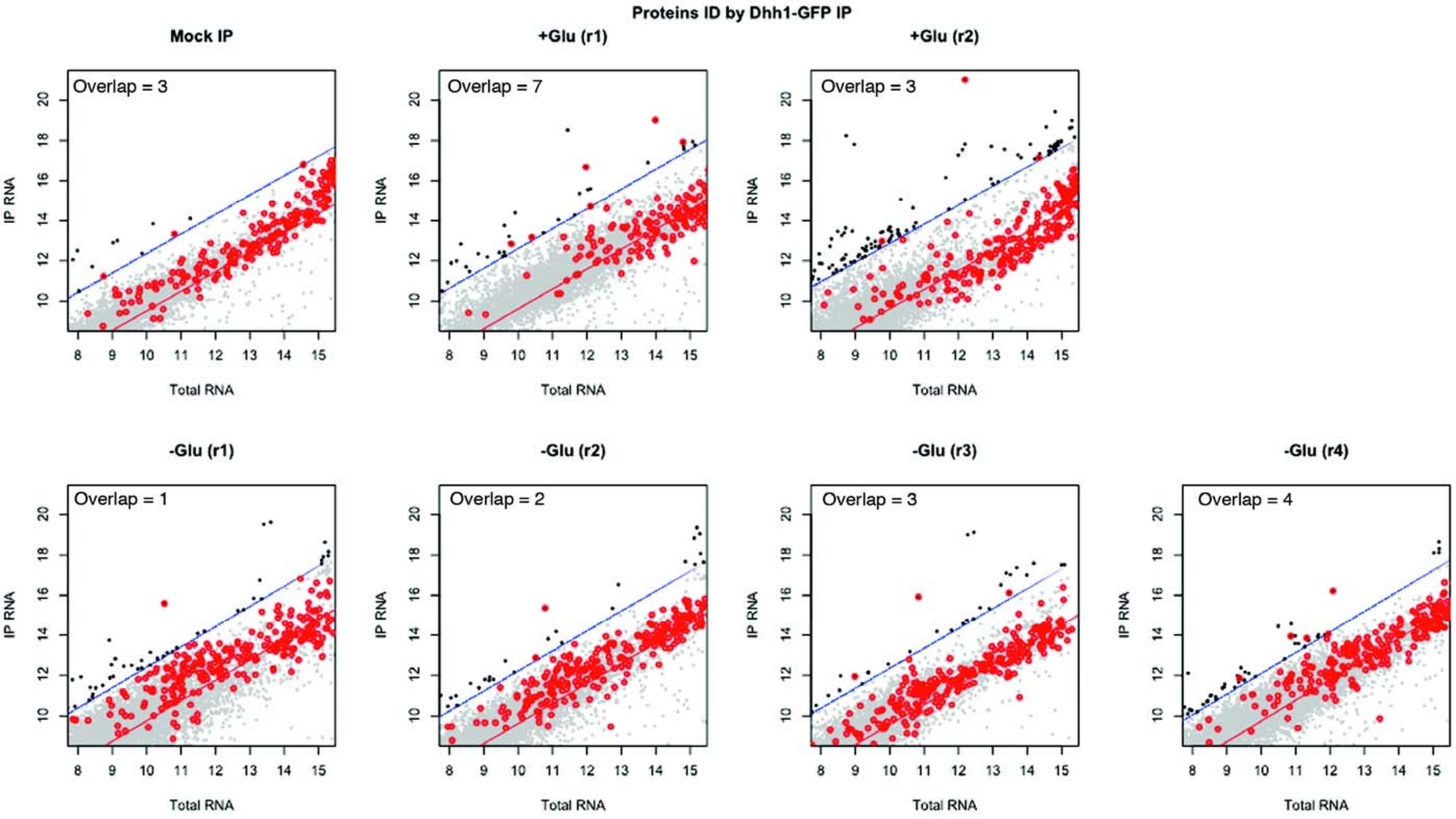

Figure S6. Protein and RNA co-enrichment. The transcripts encoding the 329 proteins identified in the proteomics dataset are circled in red. For each immuno-isolation of Dhh1-GFP, the overlap between enriched transcripts and the total number of all proteins identified is reported.
