# Supplementary material for: Proteomic Analysis of Dhh1 Complexes Reveals a Role for Hsp40 Chaperone Ydj1 in Yeast P-Body Assembly
Source: G3 (Bethesda). 2015 Sep 21;5(11):2497–511. doi: 10.1534/g3.115.021444 (PMC4632068; doi:10.1534/g3.115.021444)
Supplement: Supporting Information [file supp_g3.115.021444_TableS1.pdf]

Table S1. *S. cerevisiae* strains used in this study.

Strain	Genotype	Reference
BY4741	MATa *his3Δ1 leu2Δ0 met15Δ0 ura3Δ0*	(Winston *et al.* 1995)
YAD49	MATa *his3Δ1 leu2Δ0 met15Δ0 ura3Δ0 DHH1-GFP::HIS3MX6*	(Huh *et al.* 2003)
YAD50	MATa *his3Δ1 leu2Δ0 met15Δ0 ura3Δ0 EDC3-GFP::HIS3MX6*	(Huh *et al.* 2003)
YAD52	MATa *his3Δ1 leu2Δ0 met15Δ0 ura3Δ0 LSM1-GFP::HIS3MX6*	(Huh *et al.* 2003)
YAD53	MATa *his3Δ1 leu2Δ0 met15Δ0 ura3Δ0 PAT1-GFP::HIS3MX6*	(Huh *et al.* 2003)
YAD557	MATa *his3Δ1 leu2Δ0 met15Δ0 ura3Δ0 DHH1-GFP::HIS3MX6 ydj1Δ::kanMX*	This study
YAD591	MATa *his3Δ1 leu2Δ0 met15Δ0 ura3Δ0 DHH1-GFP::HIS3MX6 hsp104Δ::kanMX*	This study
YAD553	MATa *his3Δ1 leu2Δ0 met15Δ0 ura3Δ0 DHH1-GFP::HIS3MX6 ssa1Δ::kanMX*	This study
YAD554	MATa *his3Δ1 leu2Δ0 met15Δ0 ura3Δ0 DHH1-GFP::HIS3MX6 ssa2Δ::kanMX*	This study
YAD556	MATa *his3Δ1 leu2Δ0 met15Δ0 ura3Δ0 DHH1-GFP::HIS3MX6 hsp82Δ::kanMX*	This study
YAD555	MATa *his3Δ1 leu2Δ0 met15Δ0 ura3Δ0 DHH1-GFP::HIS3MX6 hsc82Δ::kanMX*	This study
YAD559	MATa *his3Δ1 leu2Δ0 met15Δ0 ura3Δ0 LSM1-GFP::HIS3MX6 ydj1Δ::kanMX*	This study
YAD561	MATa *his3Δ1 leu2Δ0 met15Δ0 ura3Δ0 EDC3-GFP::HIS3MX6 ydj1Δ::kanMX*	This study
YAD393	MATa *his3Δ1 leu2Δ0 met15Δ0 ura3Δ0* + p413-TEF-GFP(S65T)	This study
